# Rare Reported Left Hepatic Subcapsular Biloma and Management

**DOI:** 10.1155/2017/8609185

**Published:** 2017-07-17

**Authors:** Sarah Brown, Pablo Giuseppucci, Christopher Esper

**Affiliations:** Department of Surgery, University of Pittsburgh Medical Center, Pittsburgh, PA, USA

## Abstract

Subcapsular bilomas are a rare complication of laparoscopic cholecystectomy and an even more rare occurrence to occur spontaneously. We present a case of left sided subcapsular biloma following a laparoscopic cholecystectomy. The location of the biloma was unrelated to our area of dissection. The operation was without difficult dissection or pressurization of the biliary tree. In addition, we present percutaneous drainage alone, without ERCP as adequate management in subcapsular bilomas.

## 1. Introduction

Bilomas are loculated collections of bile that may develop due to iatrogenic causes, traumatically, or spontaneously with biliary tree disruption. Most commonly, bilomas occur in the extrahepatic space with relatively few instances of hepatic subcapsular bilomas. On literature review, bilomas are often speculated to be secondary to pressurization of the biliary tree such as post-ERCP or intraoperative cholangiogram [1–5] or following cholecystectomy with a particularly difficult dissection [6] most commonly of the right liver. Thus we present a rare case of a 72-year-old female who develops a left sided hepatic subcapsular biloma status after an uncomplicated laparoscopic cholecystectomy.

## 2. Case Report

**Figure 1:**
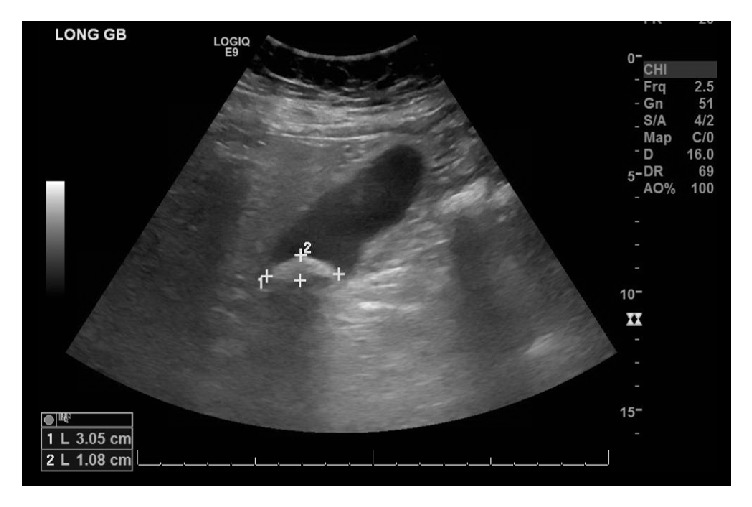
Preoperative ultrasound shows cholelithiasis.

**Figure 2:**
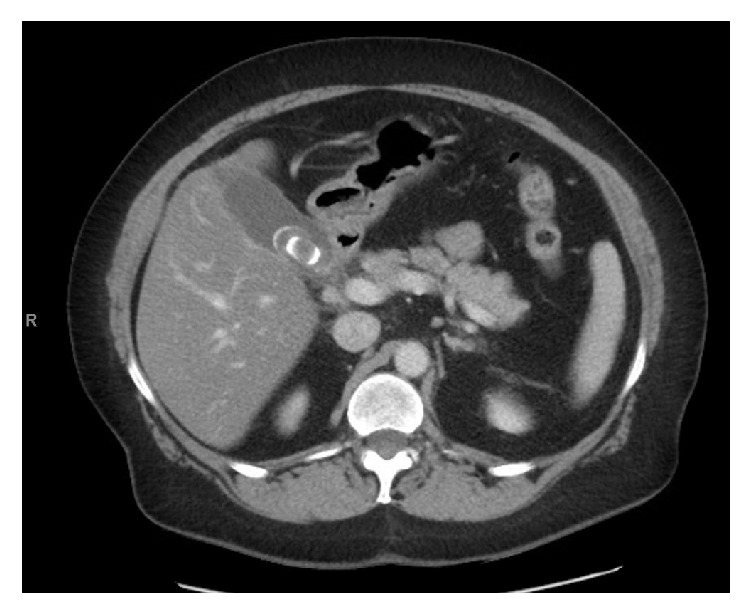
Preoperative CT abdomen with cholelithiasis and duodenal wall thickening.

**Figure 3:**
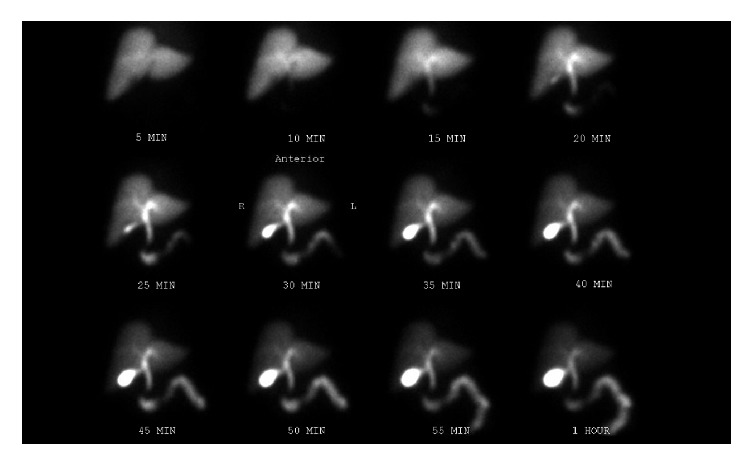
Preoperative HIDA scan shows normal filling.

A 72-year-old female was admitted with a known history of cholelithiasis and a one-day history of epigastric abdominal pain. Preoperative bloodwork revealed a leukocytosis of 15,200 cell/*µ*L; all other blood tests including CMP were within normal limits. Ultrasound demonstrated a gallstone measuring 1 cm by 3 cm and some fatty liver changes however no evidence of wall thickening, pericholecystic fluid, or ductal dilation (Figure 1). CT abdomen and pelvis with IV contrast demonstrated cholelithiasis as well as thickening of the duodenum and fluid adjacent to the pancreas (Figure 2). Physical exam demonstrated tenderness to palpation in the epigastric region without positive Murphy's sign or other peritoneal signs. Given the clinical concern for acute cholecystitis, a HIDA scan was performed that demonstrated normal filling of the gallbladder as well as spillage into the small bowel (Figure 3). However due to continued unremitting abdominal pain and leukocytosis, a laparoscopic cholecystectomy was performed under general anesthesia. On laparoscopy, there was evidence of some purulent drainage in the right upper quadrant; otherwise there was no evidence of acute inflammation of the gallbladder, pericholecystic fluid, adhesions, or gallbladder perforation. Additionally, there was no alternative evidence on exploration for the purulent fluid in the right upper quadrant. Laparoscopic cholecystectomy was otherwise unremarkable. Pathology demonstrated mild chronic cholecystitis and cholelithiasis with prominent serositis of uncertain origin.

**Figure 4:**
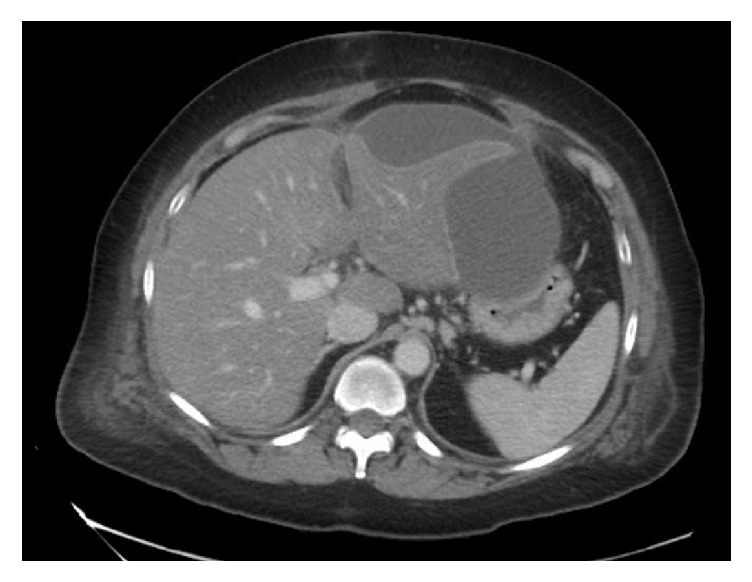
CT abdomen demonstrating subcapsular collection.

**Figure 5:**
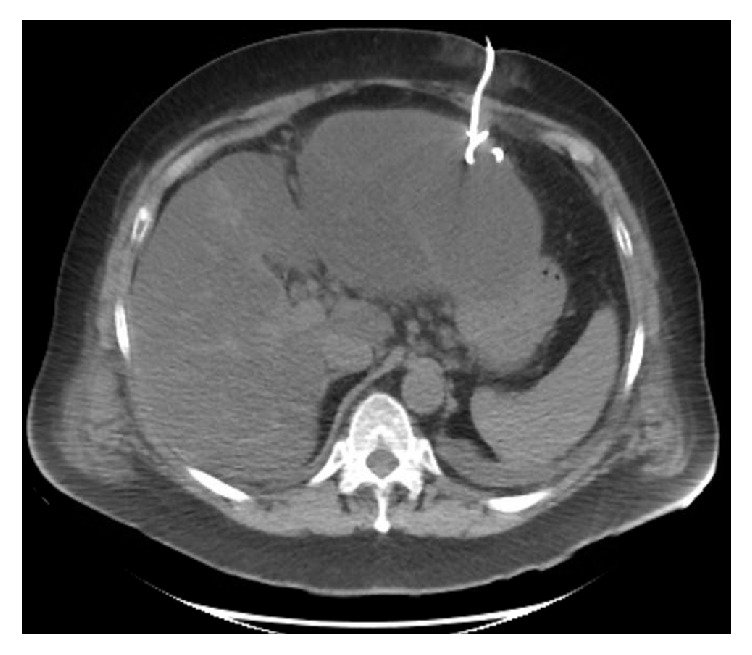
CT guided percutaneous drainage of subcapsular collection.

**Figure 6:**
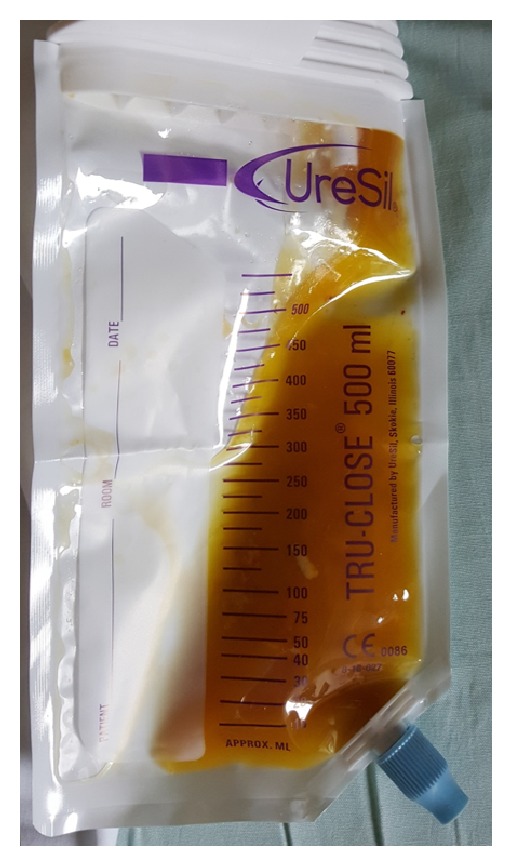
Percutaneous pigtail catheter drainage.

During her postoperative course, the patient's pain from admission resolved with improvement of her leukocytosis to 12,800 cell/*µ*L. The patient met discharge criteria and was discharged home in stable condition on postoperative day 1. The patient returned to the ER on postoperative day 4 with complaint of recurrent epigastric abdominal pain, nausea, and emesis. Bloodwork demonstrated a leukocytosis of 23,400 cell/*µ*L with new mildly elevated AST and ALT 196 u/L and 431 u/L, respectively. Total bilirubin was within normal limits at 1 mg/dL. There was no evidence of obstructive jaundice on exam or on routine lab work. CT abdomen and pelvis with IV and oral contrast revealed a small subhepatic fluid collection unsurprising in light of recent surgery; however there was additionally a new subcapsular fluid collection measuring approximately 15 HU (Figure 4). There was no other evidence of intra-abdominal inflammation or fluid collections seen on CT imaging. Given radiographic findings and clinical picture, the patient was readmitted to the hospital and started on broad spectrum antibiotics, conservatively managed for 2 days. Despite conservative management, the patient suffered from abdominal pain with persistent leukocytosis. The decision was made to radiographically drain the subcapsular fluid collection. An 8.5 Fr pigtail drainage catheter was inserted under local anesthesia under CT guidance by interventional radiology (Figure 5). Over a liter of bilious fluid was drained upon placement (Figure 6) and 100 cc the following day, which gradually deceased until there was no drainage from the pigtail catheter. Urine culture from the second admission as well as subcapsular fluid cultures both grew Pseudomonas aeruginosa. Blood cultures obtained on both the first and second admission were negative. The catheter was therefore maintained and the patient was discharged home with long term antibiotics. Repeat CT demonstrated resolution of the biloma and the pigtail catheter which was removed on follow-up at 3 weeks. On follow-up, the patient reported resolution of all abdominal pain and was recovering well.

## 3. Discussion

While bile leak is a known complication after laparoscopic cholecystectomy, the overall rate of bile duct injuries (Table 1) is approximately 0.82% with 0.39% being cystic duct leak or biliary peripheral radical which are Amsterdam Classification Type A [7, Table 1]. More commonly bile leaks occur into the peritoneal cavity; rarely according to literature review did bile leaks occur into subcapsular space. In two cases the patient undergone laparoscopic cholecystectomy with intraoperative cholangiogram prior to developing a subcapsular biloma and in one instance following an endoscopic retrograde cholangiopancreatography both of which pressurized the biliary duct system [1, 3, 5]. Other cases, both laparoscopic and open cholecystectomies with difficult dissections were complicated by bile leak thought to be caused by injury to a biliary duct radical or requiring additional manipulation with common bile duct exploration [2, 4, 6].

The cause of this patient's left sided hepatic subcapsular biloma is hypothesized as either spontaneous given the location of the biloma or iatrogenic given recent laparoscopic cholecystectomy four days prior to presentation. The location of the subcapsular biloma on the left side of the liver was far away from our area of operation and left sided subcapsular biloma is thus far unreported according to our literature review following intervention. It should also be noted in this patient's case that there was no difficult dissection, pressurization of the biliary tree by cholangiography, or even Mirizzi syndrome due to cholelithiasis. A limitation of our case presentation is that while we have both the urine culture and subcapsular biloma fluid culture confirming the presence of Pseudomonas aeruginosa with the same sensitivities and therefore a likely source, there however was no culture obtained intraoperatively of the purulent fluid in the right upper quadrant. The serositis noted on pathology as an alternative cause for the patient's symptoms is a possibility rather than cholelithiasis and uneventful laparoscopic cholecystectomy. In the instance of the infected biloma, the likely source was seeding from the urine, despite negative blood cultures.

We also present percutaneous drainage of subcapsular biloma as a viable management option. The risk of rupture of the subcapsular biloma and resulting bile peritonitis necessitates drainage. Given the appearance of the drainage in this case, JP to serum total bilirubin ratio >5 helped confirm our diagnosis and guide our management. Darwin et al. established in a small prospective case series that a JP drain fluid-to-serum bilirubin ratio of 5 or more was 100% sensitive and specific in bile leaks [8]. In this patient's case, the percutaneous drainage resulted in resolution of her abdominal symptoms. Prior to discharge, the percutaneous drain output stopped. Discussion was held that should the drainage persist, ERCP for decompression of the biliary tree would have been pursued. However further intervention was not required, the patient being adequately managed with percutaneous drainage alone as first line treatment. The percutaneous drain was left in place on discharge due to the positive fluid cultures for Pseudomonas aeruginosa. Similar to other cases, percutaneous drainage without ERCP is demonstrated to be an effective method of biloma evacuation.

## Figures and Tables

**Table 1:** Amsterdam classification of bile duct injuries.

Type	Specification
A	Leakage from cystic duct or peripheral radicals
B	Major bile duct injury with leakage
C	Bile duct stricture without leakage
D	Complete transection or excision of common bile duct
